# High abdominal fat deposition was regulated by abdominal fatty acid synthesis related genes and reduced reproduction in late stage of Chinese indigenous Wannan yellow chicken

**DOI:** 10.5713/ab.250869

**Published:** 2026-04-28

**Authors:** Hailiang Yu, Lei Wu, Hao Wang, Chenxu Yuan, Jiajia Shi, Qianyun Ji, Yaru Bai, Xiaoli Zhu, Liping Guo, Cheng Zhang, Xingyong Chen

**Affiliations:** 1 College of Animal Science and Technology, Anhui Agricultural University, Hefei, China; 2 Anhui Province Key Laboratory of Local Livestock and Poultry Genetic Resource Conservation and Bio-breeding, Anhui Agricultural University, Hefei, China; 3 Anhui Huadong Mountain Fresh Agricultural Development Co., Ltd., Xuancheng, China

**Keywords:** Abdominal Fat Thickness, Colored Ultrasound, Egg Production, Fatty Acid Synthesis, Wannan Yellow Chicken

## Abstract

**Objective:**

Indigenous chicken breeds have poorer reproductive performance than commercial laying hens. This study aims to elucidate the impact of abdominal fat deposition on the reproductive performance of indigenous chickens, providing a theoretical basis for the breeding of high-yielding chickens.

**Methods:**

A pure-bred dam line selected from Wannan yellow chickens at 60 wk of age was used to measure abdominal fat thickness using colored ultrasound. The hens were divided into two groups: high abdominal fat thickness (HF) and low abdominal fat thickness (LF). Egg production was recorded individually and compared in two connective generations. The concentrations of blood lipid, hatchability, and the expression of lipid synthesis-related genes in various tissue were examined.

**Results:**

The abdominal fat and skin thickness were significantly greater in the HF group than that of the LF group. At 60 wk of age, the egg production of the LF group was significantly higher than that of the HF group in the two generations. Abdominal fat thickness ranging from 0.4 to 1.0 cm was associated with the highest egg production. The rate of healthy embryos was significantly reduced in the HF group. Serum VLDL was significantly higher in HF than that of the LF at all detection stages. The expression of genes related with fatty acid synthesis showed no significant differences between the liver and ovary in the two groups. However, the expression of *ACACB*, *ITGAV* and *ZC3H12A* was significantly lower in HF group than that of the LF group.

**Conclusion:**

The increased abdominal fat thickness of Wannan yellow chickens resulted in decreased egg production and hatchability during the late laying period (60 wk). Genes associated with fatty acid synthesis are expressed in abdominal fat, rather than in the liver, responsible for fat deposition in abdomen at the late 60 wk of age in chickens.

## INTRODUCTION

Fat can be deposited at different sites in chicken and the most focused sites are abdominal fat, subcutaneous fat and intramuscular fat [1]. Fat deposition could be varied with tissue types and age [2,3]. Abdominal fat deposition could be largely increased during aging, especially after 40 wk of age, in which the egg production and metabolism are both decreased [4]. It is obvious that excessive fat deposition in abdomen could reduce feed efficiency and egg production performance [5]. Chicken abdominal fat has a higher heritability rate (0.82) than that of live body weight (0.55) and body parts of thighs (0.31), drumsticks (0.51), and breast muscle (0.55) at the age of selection [6]. Therefore, genetically individual selection seems to be the most efficient way to reduce abdominal fat deposition due to its high heritability. While the direct selection of abdominal fat was used by sib-test procedures, which exhibited low accuracy and time-consuming [7]. Indirect selection against abdominal fat was used by measuring plasma concentration of very low-density lipoproteins [8], which changed greatly with age, feed, and nutrient level. With the development of ultrasound technology, direct measurement of abdominal fat thickness by ultrasound could become the most convenient way for the abdominal fat selection.

Breeders selected for high or low abdominal fat in meat-type chickens showed no significant difference in egg production, while the egg weight was significantly lowered in broiler hens with high abdominal fat [9]. Mohiti-Asli et al [10] found that the egg-laying performance, fertility index and hatchability of overfed hens were significantly lower than those of lean hens on a restricted diet. Therefore, abdominal fat deposition affects the reproductive performance of chickens.

The high heritability suggested that abdominal fat deposition is mainly genetically regulated. Fatty acids are 90% synthesized in liver through many signal pathways including fat metabolism which involves acetyl-coA carboxylase alpha (ACACA) and fatty acid synthase (FASN) in the fatty acid metabolism pathway [11,12]. There is still less than 10% fatty acid could be synthesized in adipose tissue which involves acetyl-coA carboxylase beta (ACACB), integrin subunit alpha V (ITGAV), and zinc finger CCCH domain-containing protein 12a (ZC3H12A) [13,14].

In the pursuit of high-quality poultry meat, yellow-feathered chickens, especially selected from indigenous breed, has been widely used in meat-type chicken breeding system. In this study, the S3 line selected from Wannan yellow chicken, a Chinese indigenous breed, was used as experimental model. The abdominal fat and skin thickness were measured *in vivo* through B-ultrasound. The effects of abdominal fat on reproduction performance were explored by evaluating the hatchability, egg production, slaughter performance and blood lipids. A model was constructed to evaluate the optional abdominal fat thickness. The expression level of genes related to fatty acid synthesis was determined in liver and abdominal fat tissues. The results could provide a theoretical and practical basis for improving the reproductive performance of indigenous chickens.

## MATERIALS AND METHODS

### Experimental population

A pure-bred dam line selected from Wannan chickens, a Chinese indigenous breed provided by Anhui Huadong Mountain Fresh Agricultural Development, at their 60 wk of age (1,200 hens at their F8 generation) was used for abdominal fat thickness measurement by colored ultrasound (P60-VET; Jiangsu Dawei Medical). The highest and lowest 10% individuals of 60 hens in each were selected according to the thickness of abdominal fat and were assigned to high (HF) or low (LF) abdominal fat group with 6 birds in each group. Artificial insemination technology was used to obtain fertilized eggs. Eggs were collected from the two groups for incubation. Egg production was calculated from the individual records. After wing-vein blood collected from randomly selected 20 birds with 10 in each group for serum lipid determination, the birds were all slaughtered for liver, abdominal fat and ovary collection for RNA extraction. The offspring of these 60 birds were traced with their serum lipid content at their 12, 20 and 28 wk of age (10 birds at each wk of age in each group), abdominal fat and skin thickness at 28, 40 and 60 wk of age (396 birds in total), and egg production from 21 to 60 wk of age. The flow of experimental design is shown in Figure 1.

### Measurement of abdominal fat and skin thickness

Abdominal fat and skin measurement was located at the middle of the lower end of sternum and cloaca (Figure 2). The skin exposed between feathers was covered with coupling agent, and the thickness of abdominal fat and skin was measured by colored ultrasound instrument with wavelength of 7.5MHz (P60-VET; Jiangsu Dawei Medical).

### Egg production performance

Individual egg production was recorded by using a scanner that connected with breeding system (Liyuan breeding software 1.0) and scanning the QR code on each cage. The egg production performance in selected chickens (F8 generation) and their offsprings were both recorded till 60 wk of age (F9 generation).

### Blood lipid determination

Blood was collected from wing vein of chickens after 12 h fasting. The collected blood was centrifuged at 3,000×g for 10 min and the supernatant was collected for total cholesterol (CHOL), triglyceride (TG), high density lipoprotein cholesterol (HDL-C) and low-density lipoprotein cholesterol (LDL-C) determination by auto-biochemical analyzer (TBA-120R; Toshiba). The content of very low-density lipoprotein cholesterol (VLDL-C) was calculated by total CHOL minus HDL-C and LDL-C.

### Reproductive performance

During incubation, egg fertility rate, Death rate, hatchability, and the number of healthy chickens was monitored, and their ratios were calculated using the following methods. Fertility rate (%) = (Fertilized eggs / Eggs put into incubation) × 100%. Eggs with blood rings or blood lines are counted as fertilized eggs, and eggs with broken yolks are counted as unfertilized eggs. Death rate (%) = (Number of dead embryos / Number of fertilized eggs) × 100%. Hatchability (%) = (Number of hatched chicks / Number of eggs put into incubation) × 100%. Healthy rate (%) = (Number of healthy chicks / Number of hatched chicks) × 100%. Healthy chicks refer to chicks with good mental condition, glossy feathers, well-developed navels without deformities, and active behavior.

### Real-time quantitative polymerase chain reaction

Total RNA was extracted from liver, abdominal fat and ovary by Trizol Reagent (Shanghai Yisheng Biotechnology). The quality and quantity of RNA samples were determined using Nanodrop2000 spectrophotometer (Thermo Fisher Scientific), and the value of OD260/280 within 1.8 to 2.0 was considered qualified. The cDNA synthesis was performed using the PrimeScript RT Reagent Kit with the gDNA Eraser Kit (HiScriptIII 1st strand cDNA Synthesis kit). Quantitative polymerase chain reaction (qPCR) was performed by using SYBR Green Master Mix (Low Rox Plus) from Hifair, Shanghai Yisheng Biotechnology, on a cycler (ABI 7500). The reaction system was 10 μL, including 5.0 μL Hieff qPCR SYBR Green Mix, 0.2 μL upstream primer and 0.2 μL downstream primer, 1 μL total RNA template, and 3.6 μL RNase free ddH2O. The reaction conditions were set as follows, 95°C for 5 min, followed by 40 cycles (95°C for 10 s, 60°C for 20 s, and 72°C for 20 s), and then 95°C for 15 s, 60 for 1 min and 95°C for 15 s. The β-actin was set as reference gene; All primers used for relative gene expression were designed by Primer Premier 5 software according to the gene sequence in NCBI and listed in Table 1.

### Distribution of *ITGAV* gene polymorphism

Based on the chicken *ITGAV* gene sequence provided in GenBank (accession number: NC 019460.2), ITGAV gene-specific primers were designed using Primer Premier 5.0 software (primer information is shown in Supplement 1). Primers were synthesized by Sangon Biotechnology (Shanghai). PCR amplification was performed using chicken tissue DNA, and the product was sent for Sanger sequencing.

### Association analysis

The association between different genotypes of the mutation sites and reproductive traits was analyzed using the general linear model (GLM). The linear model is as follows:


(1) y=u+Age+G+e

In the formula, y represents the phenotypic value; u represents the population mean; Age represents the age of the chickens (fixed effect); G represents the genotype effect (random effect); e represents random error.

### Statistical analysis

The lipid content, abdominal fat and skin thickness, egg production, incubation and gene expression level were compared between high and low abdominal fat groups by independent sample t-test using SAS9.3. Egg production among chickens with different abdominal fat thickness was analyzed by one way ANOVA (SAS 9.3 edition) followed by Duncan’s multiple comparison test. The correlation between abdominal fat thickness and egg production was analyzed by Pearson correlation using SAS9.3. Popgen32 software was used to calculate locus heterozygosity (He), effective number of alleles (Ne), allele frequency, and genotype frequency. Linkage disequilibrium and haplotype analysis of single nucleotide polymorphism (SNP) loci were performed using the online software SHEsis. All production-related test data for each group are expressed as mean±standard deviation.

## RESULTS

### Comparison of skin and abdominal fat thickness between HF and LF groups

According to the observed image from B-ultrasound (Figure 3), the outline of each tissue is clearly presented, and the first part (marked 1) of the top layer was the thickness of skin, and the second part (marked 2) was the thickness of abdominal fat. The thickness of abdominal fat was significantly different among individuals. According to the thickness of abdominal fat, the chickens was divided into high abdominal fat group and low abdominal fat group.

Comparison of the thickness of abdominal fat and skin in F8 generation (Table 2), the thickness of abdominal fat from HF group was significantly higher and was 3.7 times than that of LF group at 60 wk of age (p<0.05). In addition, the skin thickness was also significantly higher in HF group than that of LF group at 60 wk of age (p<0.001).

The skin thickness in F9 generation showed no significant difference at 40 and 60 wk of age (Table 2, p>0.05). However, the abdominal fat thickness was significantly higher in HF group than that of the LF group at 60 wk of age (Table 2, p<0.001).

### Effect of abdominal fat thickness on hatchability

To reveal the effect of abdominal fat thickness on hatchability, the hatchability data from F8 generation chickens with high or low abdominal fat thickness was compared (Table 3). The embryo death rate was significantly higher in HF group than that of LF group (p = 0.035). The healthy rate was significantly higher in LF group than that of HF group (p = 0.039). The fertility rate and hatching rate showed no significant difference in the two groups (p>0.05).

### Comparison of blood lipids content in chickens with high and low abdominal fat thickness

Then, we compared the blood lipids content in chickens with high and low abdominal fat thickness. The results showed that blood lipids of CHOL, TG, LDL-C, HDL-C showed no significant difference in chickens with high and low abdominal fat thickness at F8 generation and their offspring F9 generation (Figures 4A, 4C, 4D, 4E, p>0.05). The lipid content of VLDL-C was significantly higher in chickens with high abdominal fat thickness than that of the low abdominal fat thickness at F8 generation and their offspring F9 generation, with the highest VLDL-C concentration at 28 wk (Figure 4B, p<0.05).

### Effect of abdominal fat thickness on egg production performance

To explore whether abdominal fat thickness influences chickens’ laying performance, the egg production performance was compared in chickens with high or low abdominal fat thickness in two connective generations of F8 and F9 (Table 4). Egg production performance showed no difference between LF and HF groups before 40 wk of age in the two generations (p>0.05). Interestingly, chickens 60 wk egg production performance was significantly higher in LF groups as compared with that of the HF groups in the two generations (p<0.05).

### The relationship between abdominal fat thickness and egg production performance

Furthermore, we conducted correlation analysis to reveal the relationship between abdominal fat thickness and egg production performance (Supplement 2). The results suggested that an increased abdominal fat thickness was associated with a higher skin thickness (r = 0.296, p<0.001), and a lower egg production performance (r = −0.1000, p<0.05). The skin thickness was significantly negatively correlated with egg production. (r = −0.204, p = 0.015).

The abdominal fat thickness was artificially divided into 8 groups with 0.2 cm intervals and the number of egg production was calculated within each interval of group (Table 5). The number of egg production was significantly different among groups, and was exhibited the highest in chickens with the abdominal fat thickness of 0.4–0.6 cm and 0.8–1.0 cm (Table 5). While chickens with low abdominal fat thickness of 0.2–0.4 cm or high abdominal fat thickness of larger than 1.7 cm produced 199.84 and 198.5 eggs, respectively, and showed the lowest number of egg production. Therefore, a multiple linear regression model of abdominal fat thickness and egg production was constructed, and the relationship between abdominal fat thickness and egg production could be expressed as y = 205.95+15.86x−10.53x^2^, where y is the number of egg production, x is the abdominal fat thickness.

### Effect of abdominal fat thickness on genes expression level at different tissues

In considering the specific expression of genes in each tissue, the expression of *FASN* and *ACACA* was detected in liver tissue, the expression of *ACACB*, *ITGAV* and *ZC3H12A* was detected in abdominal fat tissue, while the expression of *ITGAV*, *FSHR* and *ZC3H12A* was detected in ovarian tissue of F8 generation chickens at 60 wk of age. The expression of *FASN* and *ACACA* in liver was digitally higher in chickens with low abdominal fat thickness (Figure 5A), however, no significant differences were observed in the two groups (p>0.05). The expression of *ACACB*, *ITGAV* and *ZC3H12A* in abdominal fat was all significantly higher in chickens with low abdominal fat thickness (Figure 5B, p<0.05). The expression of *ITGAV*, *FSHR* and *ZC3H12A* in ovary was digitally lower in chickens with low abdominal fat thickness (Figure 5C), however, no significant differences were observed in the two groups (p> 0.05).

### Association between single nucleotide polymorphisms of the *ITGAV* gene and reproductive traits

SNP site detection was performed on the PCR product of the *ITGAV* gene, and the following three mutation sites were found: 7_1451836 (A>G), 7_1453027 (G>A), and 7_1451806 (C>G) (Figures 6A–6C). The results of population genetic polymorphism analysis and haplotype analysis of the *ITGAV* gene SNPs are shown in Figures 6D, 6E. All three SNPs are moderately polymorphic sites, and the first and second SNP sites are in strong linkage disequilibrium. Furthermore, at 7_1451836 (A>G) locus, the fertilization and hatching rates of the AA genotype were significantly higher than those of the GG genotype, while the fertilization rate of the AG genotype was significantly higher than that of the GG genotype (Figure 7, p<0.05). At 7_1453027 (G>A) locus, the egg production of the AA genotype was significantly higher than that of the GA genotype (Figure 7, p<0.05).

## DISCUSSION

At present, the direct selection against abdominal fat is depend on sib-test or hand palpation [7]. The uncertainty and subjectivity caused the difficult of abdominal selection. The ultrasound has been used in measuring skin and muscle thickness in duck [15]. So, a certain wavelength could also be used to measure the thickness of abdominal fat. In this experiment, the ultrasound with 7.5 MHz wavelength was used for detecting abdominal fat thickness successfully.

In poultry, the abdominal fat and subcutaneous fat are major parts of adipose tissue. An increased abdominal fat deposition in the chicken body means an increased body fat and would result in an increased subcutaneous fat. Therefore, chickens with high abdominal fat thickness also exhibited high skin thickness in this experiment. Cahaner et al [16] found that the hatchability was significantly higher by 20%–30% in low-fat line as compared with that of the high-fat line in their S2 and S3 generations, while the fertility showed no significant difference. The hatchability was higher about 8.8% in low-fat line as compared with that of the high-fat line in chickens selected for body fat at their 14th to 18th generations [17]. Leenstra [18] selected birds and established high and low- abdominal fat lines. Except for fertility rate showed no significant difference in the two lines, other hatchability was all higher in low-abdominal fat lines. Chickens without feed restriction performed higher fertility rate in low-fat line [19]. Birds in low-fat line has higher qualified offsprings and lower death rate than that of high-fat line [20]. Egg hatchability could be affected by various factors, hatching conditions, egg quality, maternal quality, etc. When the hatching condition is optimized, the egg quality that affected by maternal could be mostly important and determine the embryo development and hatchability. It is suggested that obesity caused excessive abdominal fat deposition is one of the main factors to produce low-quality oocytes [21], and thus lead to the death of embryo. Abdominal fat deposition could also press the fallopian tubes and affect ovulation, sperm storage and supply sperm to the fertilization site after ovulation, and resulted in a low egg fertility rate [22]. In this experiment, higher death embryo and low rate of qualified chickens from the high abdominal fat groups at their 60 wk of age was observed. A stronger lipid deposition in abdominal fat might result in a lower follicle development and reduced sperm combining ability.

According to the research by Hocking et al [23], the selection of low abdominal fat lines could significantly improve the reproductive performance of hens. Lean hens presented with a significantly earlier age at first egg and a higher egg number relative to fat hens [24]. These results suggest that excessive abdominal fat deposition has an adverse effect on hens, impairing their egg-laying performance. Abdominal fat accumulation needs more energy thus reduced the development of reproductive organs, affect follicle development and maturation, and reduces the quality of oocytes [25,26]. In addition, the number of atretic follicles increased in chickens from high-fat line, which directly reduced egg production. The egg production showed no significant difference before 40 wk of age between high and low abdominal fat groups in this experiment. During peak laying, the reproductive system is in high development and requires large amount of energy. The synthesized fatty acid is mainly transported to the ovary for the requirement of follicle development. Therefore, storage of certain fat could be used for yolk formation and egg production. However, at the late egg production period, the decreased egg production and enhanced fatty acid synthesis would result in deposition of abdominal fat. Therefore, a lower egg production performance existed in chickens with high abdominal fat in this experiment especially at their late egg production period. In order to balance the need of fat as energy supply for egg production, a certain amount of abdominal fat is required during egg production. In this study, an optimal abdominal fat thickness of 0.4 to 1.0 at 60 wk of age was fitted according to egg production.

During the reproduction period, large amount of VLDL is synthesized and transported from liver to ovary to meet the needs of follicle development. The concentration of VLDL and TG in the plasma is used as raw material for yolk and adipose tissue formation. Therefore, high VLDL and TG was usually observed in chicken with high lipid or in peak laying period [24,27], and VLDL was used as indirect factor for abdominal fat selection. In this experiment, the lipid concentration of VLDL was significantly higher low abdominal fat groups at 40 wk of age suggesting its high yolk precursor requirement for egg production. While a higher serum lipid VLDL was observed in high abdominal fat groups at 60 wk of age, suggested that the differential of abdominal fat deposition in chickens would be mainly expressed at the late period and then reduced reproduction in turn.

Abdominal fat deposition is mostly affected by genetic factors and its regulatory mechanism has been highly explored from molecular level. As FASN, the down expression of FASN is closely associated with less lipid deposition [28]. The key limiting enzyme of ACACA, regulates the rate of de novo fatty acids synthesis, plays an important switching role in fat metabolism and abdominal fat deposition [29]. It is stated that the expression of ACACA was increased at peak laying to satisfy the fatty acid supplementation [30]. However, the expression of *ACACA* in liver showed no significant difference in the two groups at 60 wk of age in this experiment, suggested that the high fatty acid in abdominal fat might not from liver synthesized. Inhibit the expression of acetyl-CoA carboxylase could result in the concomitant inhibition of fatty acid synthesis in rats [31]. Overexpression of *ITGAV* could seriously damage the adipogenesis, while *ITGAV* knockdown could enhance adipogenesis [13]. The expression of *ZC3H12A* was transiently activated during the transition of preadipocytes to adipocytes. The higher expression of *ACACB*, *ITGAV* and *ZC3H12A* in abdominal fat of low abdominal fat chickens suggested that abdominal fat play the additional important role for fat synthesis at 60 wk of age. However, this study has limitations. the protein expression of the genes related to fatty acid synthesis needs validation through additional research. In addition, we conducted polymorphism analysis on the *ITGAV* gene and association analysis between SNPs and reproductive traits. The results showed that both the 7_1451836 (A>G) and 7_1453027 (G>A) sites significantly affect the reproductive performance of the Wanna yellow chicken.

## CONCLUSION

Chickens before 40 wk of age showed no significant difference in abdominal fat deposition. The B-ultrasound could be used to measure the abdominal fat thickness in vivo for fat selection at 60 wk of age, and the abdominal fat thickness at 0.4 to 1.0 cm could be best for egg laying. The increased abdominal fat thickness of Wannan yellow chickens decreased the egg production and hatchability at 60 wk of age, and increased serum lipid VLDL concentration. Fatty acid synthesis related genes expressed in abdominal fat directly regulated abdominal fat deposition in chickens at 60 wk of age.

## Figures and Tables

**Figure 1:**
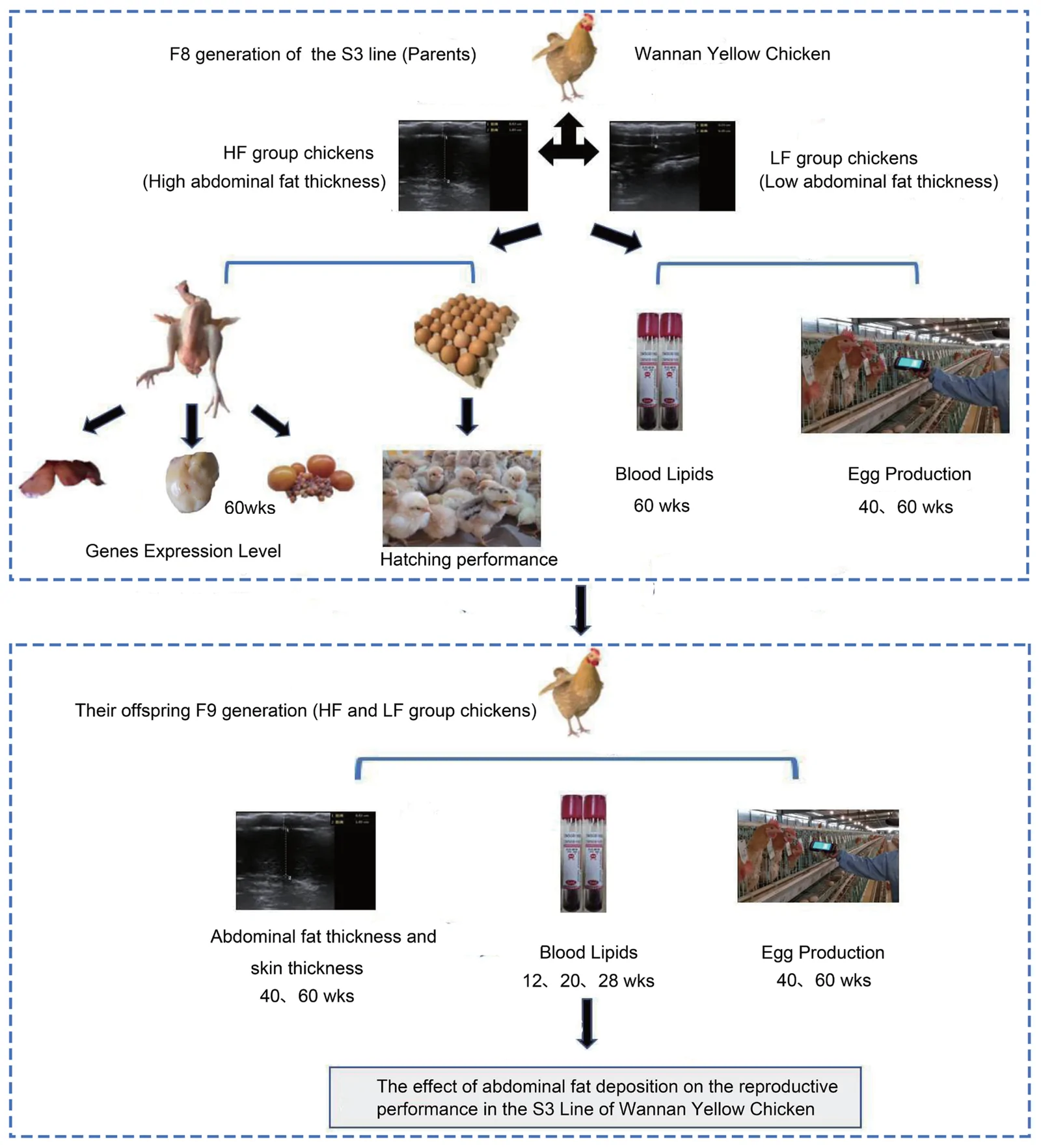
Flow diagram of experimental design. The F8 generation of Wannan yellow chickens were divided into a high abdominal fat group and a low abdominal fat group based on the abdominal fat thickness measured by colored ultrasound. The egg production performance and hatchability of the chickens were recorded. Blood lipids and tissue gene expression levels were measured at 60 wk. In the offspring of high abdominal fat and low abdominal fat chickens (F9 generation), abdominal fat and skin thickness, egg production performance, and blood lipids were measured and compared between the two groups.

**Figure 2:**
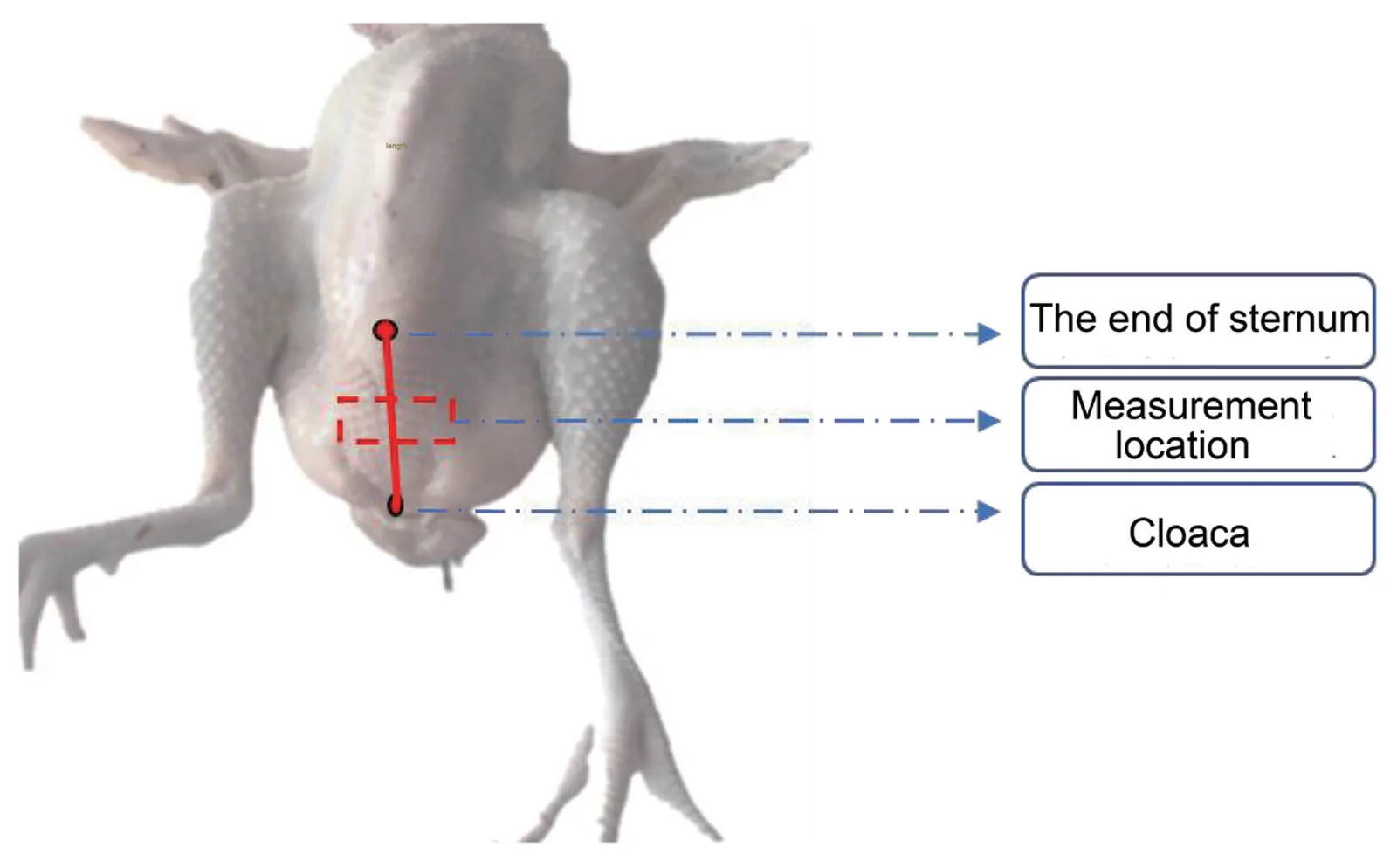
Schematic diagram of abdominal fat measurement position.

**Figure 3:**
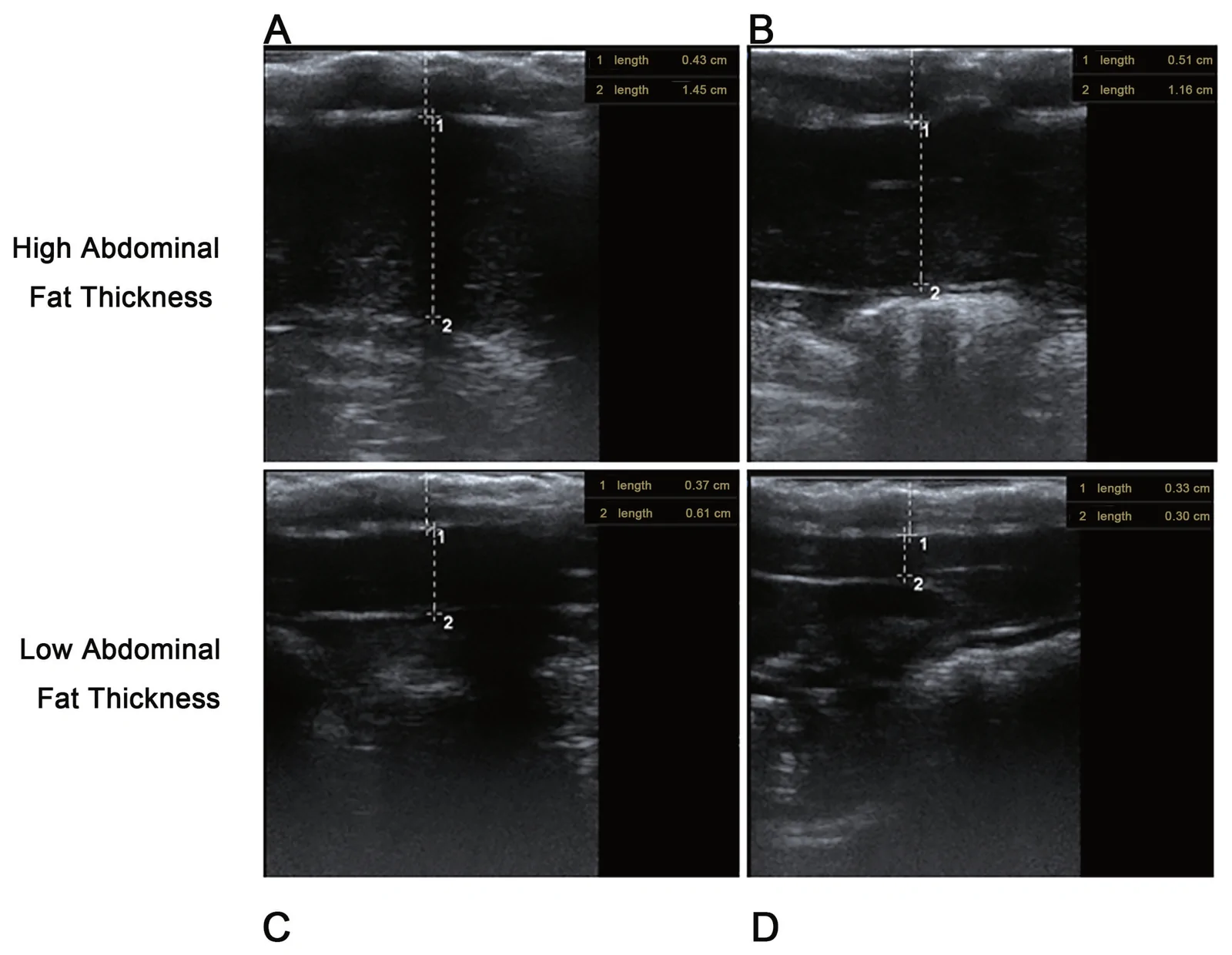
B-ultrasound imaging of abdominal fat thickness and subcutaneous thickness of Wannan yellow chicken S3 line at 60 wk of age. (A, B) Skin thickness and abdominal fat thickness in HF group chickens. (C, D) Skin thickness and abdominal fat thickness in LF group chickens. 1 represent the skin thickness and 2 represent the abdominal fat thickness. 1 and 2 in the upper right-hand corner of the picture indicate the thickness of the skin and abdominal fat, respectively.

**Figure 4:**
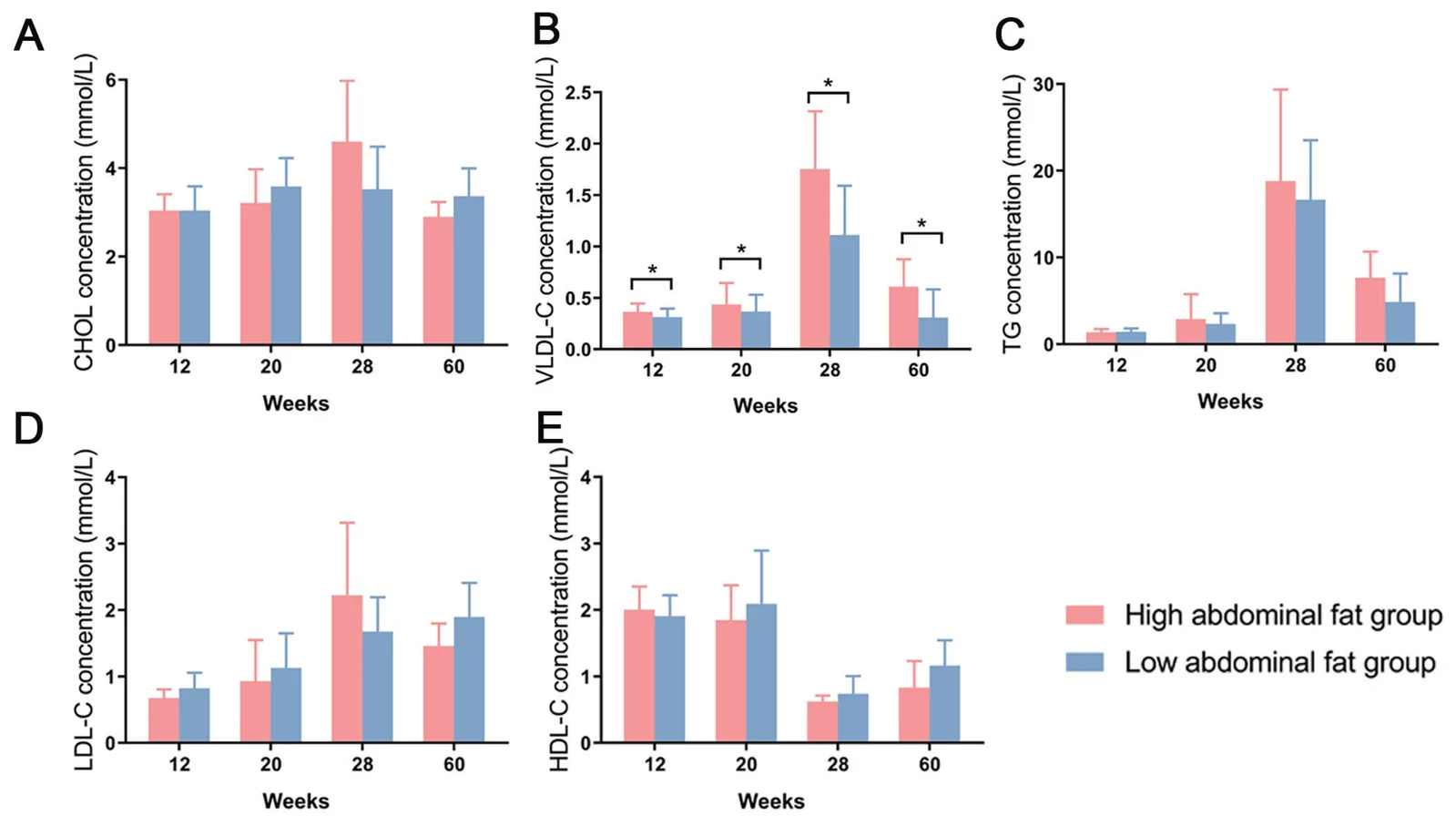
Blood lipid content at various stages in high and low abdominal fat groups of Wannan yellow chicken S3 line. The time periods selected were 60 wk of age for F8 generation chickens and 12, 20, and 28 wk of age for F9 generation chickens. (A) CHOL concentration. (B) VLDL-C concentration. (C) TG concentration. (D) LDL-C concentration. (E) HDL-C concentration (n = 10). * p<0.05. VLDL-C, very low-density lipoprotein cholesterol; TG, triglyceride; LDL-C, low-density lipoprotein cholesterol; HDL-C, high density lipoprotein cholesterol.

**Figure 5:**
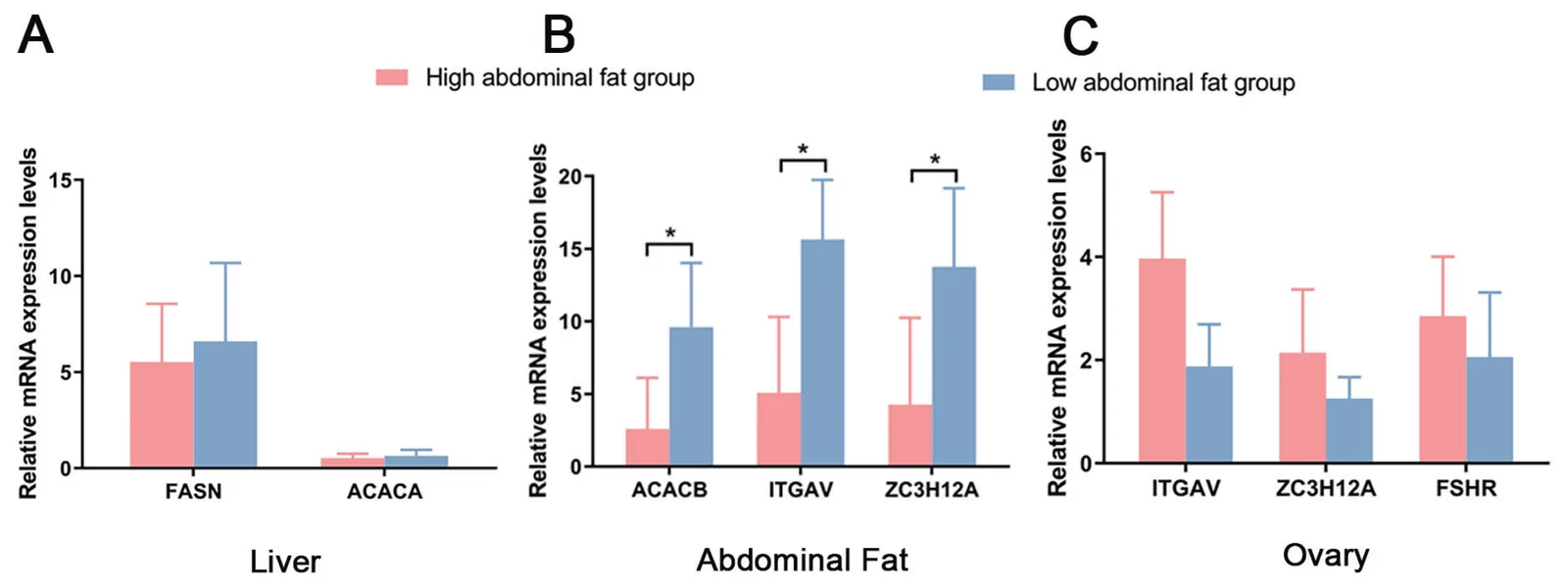
The mRNA expression of genes in liver, abdominal fat and ovarian tissue of high and low abdominal fat groups of F8 generation chickens at 60 wk of age. (A) The mRNA expression of *FASN* and *ACACA* in liver tissue (n = 10). (B) The mRNA expression of *ACACB*, *ITGAV* and *ZC3H12A* in abdominal fat tissue (n = 10). (C) The mRNA expression of *ITGAV*, *ZC3H12A* and *FHSR* in ovarian tissue (n = 10). * p<0.05.

**Figure 6:**
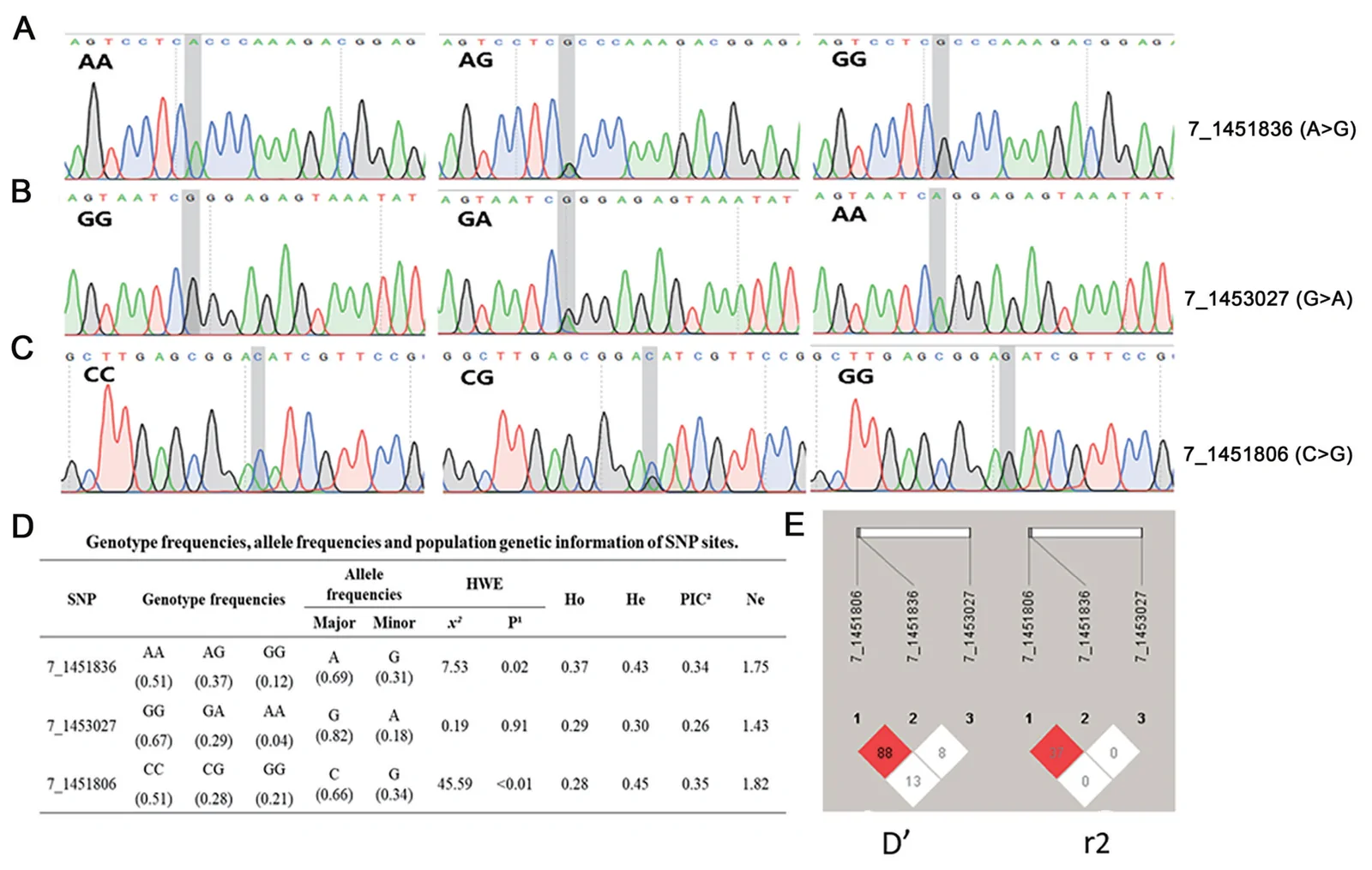
The peak plots and population genetic polymorphism analysis of the *ITGAV* gene SNPs. (A) The peak plot of the 7_1451836 (A>G) locus. (B) The peak plot of the 7_1453027 (G>A) locus. (C) The peak plot of the 7_1451806 (C>G) locus. (D) Genotype frequencies, allele frequencies and population genetic information of SNP sites. (E) The haplotype analysis of the *ITGAV* gene SNPs. SNP, single nucleotide polymorphism.

**Figure 7:**
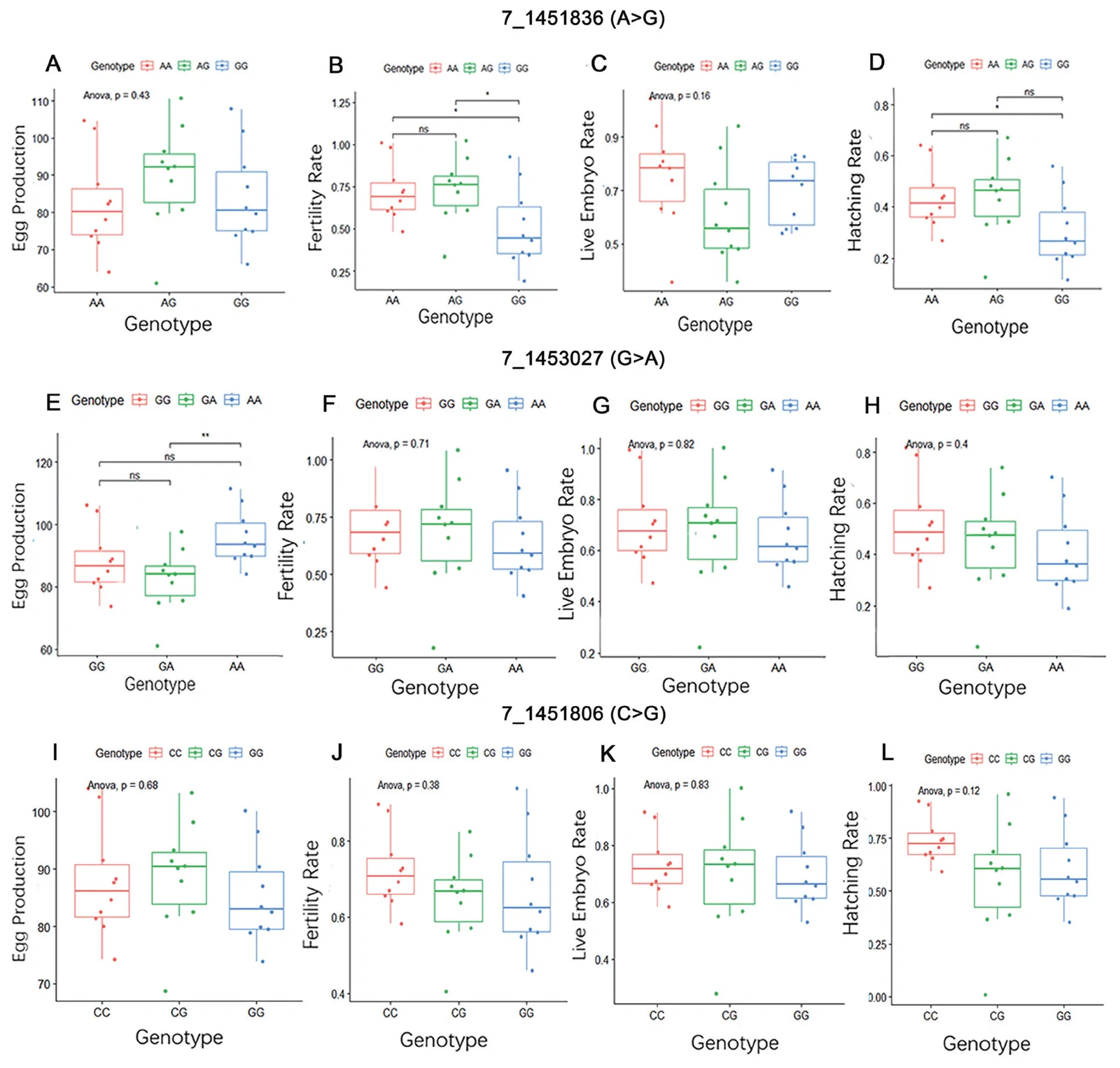
Association between SNPs of the *ITGAV* gene and reproductive traits. (A–D) Association between the 7_1451836 (A>G) locus and reproductive traits. (E–H) Association between the 7_1453027 (G>A) locus and reproductive traits. (I–L) Association between the 7_1451806 (C>G) locus and reproductive traits. * p<0.05, ** p<0.01. SNP, single nucleotide polymorphism.

**Table 1:** Primer sequences for qRT-PCR amplification of genes

Gene	Accession number	Primer sequences (5′–3′)	Product size (bp)
*ACACA*	396504	F: GCTGCCTCTGATGGTCTTTG	78
		R: AACTTGAGCACCTGGTCGTA	
*FASN*	396061	F: TTCCCACAGCTGGATGCTAA	155
		R: TGTTGAGCAAGACAACGCAA	
*ACACB*	430557	F: CAAGAACGTCCGTGAGGAAC	172
		R: TCTCTGCTGGGCAATGAGAA	
*ITGAV*	396420	F: CCATTGATGCAGATGGGCAA	197
		R: TCTGGTTGCTAACTGGTCGT	
*ZC3H12A*	419618	F: TGAAGCAGATGCCAAGGGTA	71
		R: TCTACGGCAGCTACAGAGTG	
*FSHR*	395962	F: TACCCGTCGTCCATAAGGTG	187
		R: TCATCCAGGCAGGTTCCATT	
*GAPDH*	374193	F: GTGAAAGTCGGAGTCAACGG	184
		R: CGTTCTCAGCCTTGACAGTG	

qRT-PCR, quantitative reverse transcription polymerase chain reaction.

**Table 2:** Comparative analysis of abdominal fat thickness between LF and HF groups of Wannan yellow chicken

Generation	Week of age	Abdominal fat thickness (cm)		Skin thickness (cm)	

		LF	HF	LF	HF
F8	60	0.504^b^±0.106	1.864^a^±0.150	0.284^b^±0.064	0.369^a^±0.085
F9	28	0.685±0.251	0.729±0.248	0.417±0.076	0.443±0.072
	40	0.905±0.232	1.284±0.301	0.326±0.058	0.335±0.062
	60	0.611^b^±0.124	1.763^a^±0.327	0.319±0.066	0.358±0.073

LF means chickens with low abdominal fat thickness; HF means chickens with high abdominal fat thickness.

a,b Different lowercase letters in the same row indicate significant differences (p < 0.05), and the absence of letters indicates no significant differences (p>0.05).

**Table 3:** Comparison of hatching performance between HF and LF groups of F8 generation chickens

Group	LF	HF	p-value
Fertility rate (%)	94.18±2.40	94.83±0.33	0.669
Death rate (%)	2.64^b^±1.64	6.87^a^±1.66	0.035
Hatchability (%)	88.38±3.22	86.59±2.00	0.460
Healthy rate (%)	99.69^a^±0.10	99.36^b^±0.20	0.039

LF means chickens with low abdominal fat thickness; HF means chickens with high abdominal fat thickness.

a,b Different lowercase letters in the same row indicate significant differences (p<0.05), and the absence of letters indicates no significant differences (p>0.05).

**Table 4:** Comparison of egg production performance between HF and LF groups and their next generation

Group	Egg production at F8 generation (No.)		Egg production at F9 generation (No.)	

	40 wk	60 wk	40 wk	60 wk
LF	88.23±16.02	217.51^a^±32.80	90.97±16.87	218.73^a^±28.68
HF	87.07±14.32	204.14^b^±33.04	89.72±15.35	203.12^b^±31.56
p-value	0.732	0.047	0.695	0.036

LF means chickens with low abdominal fat thickness; HF means chickens with high abdominal fat thickness.

a,b Different lowercase letters in the same column indicate significant differences (p<0.05), and the absence of letters indicates no significant differences (p>0.05).

**Table 5:** Comparison of egg production among chickens with different abdominal fat thickness

Abdominal fat thickness (cm)	0.2–0.4	0.4–0.6	0.6–0.8	0.8–1.0	1.0–1.2	1.2–1.4	1.4–1.7	>1.7	p-value
Skin thickness (cm)	0.271	0.286	0.308	0.319	0.325	0.345	0.364	0.364	<0.001
Egg production (No.)	199.84^b^	215.57^a^	206.86^ab^	217.18^a^	207.44^ab^	210.32^ab^	204.85^ab^	198.5^b^	0.038

a,b Different lowercase letters in the same row indicate significant differences (p<0.05), and the absence of letters indicates no significant differences (p>0.05).

## Data Availability

Upon reasonable request, the datasets of this study can be available from the corresponding author.

## SUPPLEMENTARY MATERIAL

**Supplement 1**. Primer sequences for PCR amplification of *ITGAV* gene.

**Supplement 2**. The correlation analysis between abdominal fat thickness, skin thickness and egg production performance in Wannan yellow chickens.
