# Supplementary material for: High abdominal fat deposition was regulated by abdominal fatty acid synthesis related genes and reduced reproduction in late stage of Chinese indigenous Wannan yellow chicken
Source: Anim Biosci. 2026 Apr 28;39(10):250869. doi: 10.5713/ab.250869 (PMC13662942; doi:10.5713/ab.250869)
Supplement: Supplementary file 1 [file ab-250869-Supplementary-1.pdf]

Supplement 1. Primer sequences for PCR amplification of *ITGAV* gene

Primer	Primer sequences (5'-3')	Product size (bp)
P1	F: AGGAAGGAGGAGCGGCATCATG<br>R: GTCAATTTGCCCTCCAAGATAA	857
P2	F: AGCCTTACTCAACGGCGTTAAT<br>R: ACATTTCAGAATTCGTGTCAGC	1009
