# Supplementary material for: High abdominal fat deposition was regulated by abdominal fatty acid synthesis related genes and reduced reproduction in late stage of Chinese indigenous Wannan yellow chicken
Source: Anim Biosci. 2026 Apr 28;39(10):250869. doi: 10.5713/ab.250869 (PMC13662942; doi:10.5713/ab.250869)
Supplement: Supplementary file 2 [file ab-250869-Supplementary-2.pdf]

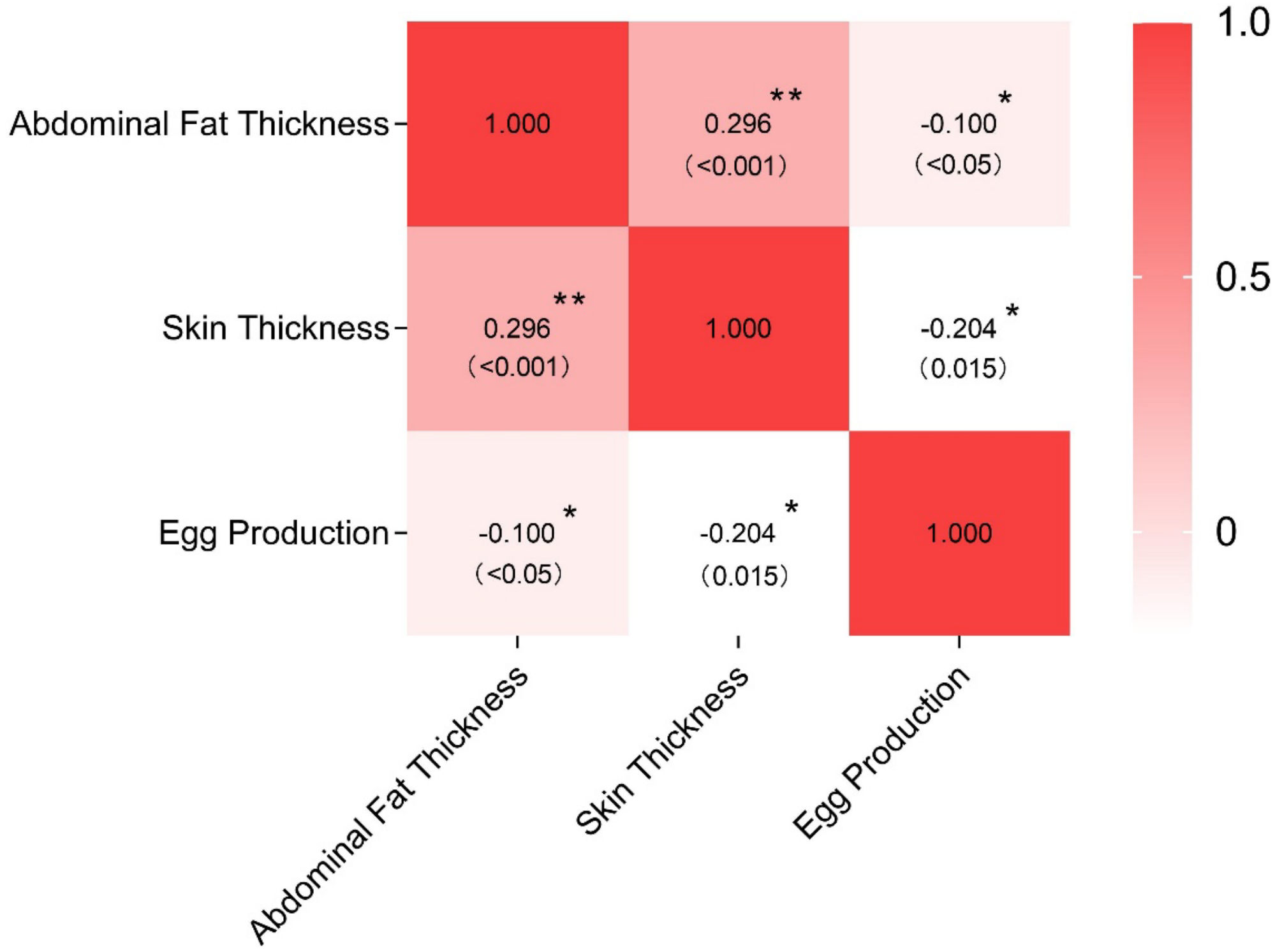

Supplement 2. The correlation analysis between abdominal fat thickness, skin thickness and egg production performance in Wannan yellow chickens. The value in the box represents the Pearson's correlation coefficient between the any two traits. The red indicates appositive correlation, and the white shows a negative correlation. * $P < 0.05$, ** $P < 0.01$.
